# Unveiling Clinical Relevance: Investigating Placentas Submitted for Histological Examination and Their Correlation with Clinical Indications and Histological Findings

**DOI:** 10.3390/life14080927

**Published:** 2024-07-24

**Authors:** Luisa Strahler, Alexander Horky, Stephan Spahn, Franz Bahlmann, Elise Gradhand

**Affiliations:** 1 Department of Pediatric and Perinatal Pathology, Dr. Senckenberg Institute of Pathology, University Hospital Frankfurt, Goethe University Frankfurt, 60590 Frankfurt, Germany; luisa.strahler@gmx.de; 2 Department Obstretrics, Bürgerhospital und Clementine Kinderhospital, 60316 Frankfurt am Main, Germany; a.horky@buergerhospital-ffm.de (A.H.); s.spahn@buergerhospital-ffm.de (S.S.); f.bahlmann@buergerhospital-ffm.de (F.B.)

**Keywords:** placenta histology, clinical information, clinicopathological correlation

## Abstract

In Germany, there is currently no official guideline for the submission of placentas for histopathological examination. Placentas are sent for histological examination by obstetricians according to locally defined indications, which leads to different practices in different centers. In this study, two cohorts of placentas were compared to assess the clinical relevance of placental examination. One cohort consisted of placentas with a clinical indication for histologic examination and the other of placentas with a clinically healthy pregnancy and a healthy infant. In this study, a placenta request form based on established international guidelines was used. Placentas from singleton and twin pregnancies with and without clinical indications were histopathologically examined. Clinical information was extracted from the request form and later correlated with histological findings. A total of 236 placentas were examined, including 127 (53.8%) with clinical indications and 109 (46.2%) without. The concordance between submission reasons and histopathological findings was higher in singleton pregnancies with clinical indications (90.9%) compared to twin pregnancies (62.97%). Placentas from singleton and twin pregnancies with clinical indications exhibited significantly more pathological findings than their respective healthy control groups. Histopathological examination of the placenta can confirm or reveal placenta pathologies and therefore improve the care of the mother, child and future pregnancies.

## 1. Introduction

The submission of placentas for histopathologic examination is already performed according to national guidelines in many countries [1,2,3,4,5,6]. These guidelines act as a triage system that lists maternal and fetal indications to determine when a placenta should be sent for histopathological examination and emphasizes the need to match histopathological findings with clinical information. For example, in the USA [4], South Australia [3] and the UK [1], guidelines for placental examination have been developed by expert groups consisting of pathologists, physicians and obstetricians. These groups have drafted request forms for placenta submission that contain information about the mother, pregnancy history and the child. The guidelines also provide detailed specifications for the transportation and histopathological processing of placentas.

In contrast, there are currently no official guidelines for sending placentas for histopathological examination in Germany. Placentas are usually sent on the basis of local indications by obstetricians, although these indications vary between centers. The lack of standardization means that placentas are often sent for histopathological examination with reluctance in Germany [7,8,9].

In other countries, studies have already shown why the histopathological examination of placentas is important [7,10,11,12,13]. It contributes to the better clarification of pregnancy complications and improves the treatment management of newborns, mothers and subsequent pregnancies [7].

However, there is not yet a study that highlights the importance of standardization in the submission and processing of placentas with clinical indication by comparing them with a healthy control group without indications. The main aim of this study is to demonstrate the importance of the standardization of the indication of the submission and histopathological examination of placentas. In Germany, there are currently no official guidelines for obstetricians on when to submit a placenta and no guidelines for pathologists on reporting placentas.

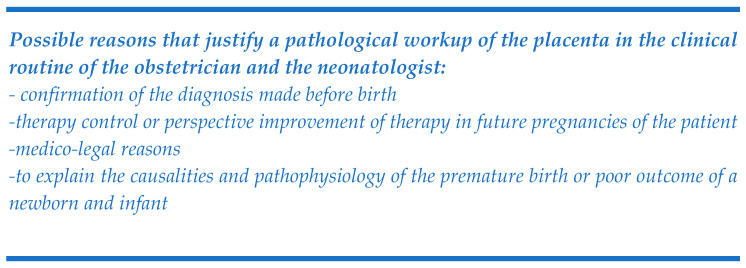



## 2. Methods

The study includes a total of 236 placentas that were examined at the Senckenberg.

Institute of Pathology at the University Hospital Frankfurt am Main between November 2019 and August 2021. The study protocol was approved by the local ethics committee UCT of the University of Frankfurt (project-number: UCT-54-2020). Of these, 127 placentas were from singleton and twin pregnancies with clinical indications and 109 placentas from singleton and twin pregnancies without clinical indications. The internal placenta request form, which is based on international guidelines, was used for the submission (see Appendix A—Clinical Request Form for Submitting the Placenta).

The histopathological workup was carried out in accordance with the departmental guidelines, which are based on the guidelines of the Royal College of Pathologists [1]. The macroscopic and microscopic examination and the preparation of a histopathology report were carried out accordingly.

The study cohort comprises a total of 236 placentas that were examined at the Senckenberg Institute of Pathology at the University Hospital Frankfurt am Main between November 2019 and August 2021. This study includes 73 pathological singleton placentas, 39 healthy singleton placentas, 70 healthy twin placentas and 54 pathological twin placentas.

The internal placenta request form, which is based on established international guidelines [1,2,3], was used to send in the placentas. This contains clinical information such as the date of delivery, gestational age, birth weight, maternal BMI, pregnancy history, APGAR and a selection for the indication of the placenta examination. For the statistical evaluation, the data were analyzed using the BiAS version 12.11-04/2022 statistics program.

The histopathological workup was performed in accordance with the institute’s internal guidelines, which are based on the guidelines of the Royal College of Pathologists [1]. The placentas were processed according to a departmental cut-up protocol adapted to international standards [1,2,4,5,6,14,15,16]. The placentas were initially examined macroscopically, whereby the completeness of the amnion membranes, the insertion and the color were assessed. The umbilical cord was examined for its length and width as well as for the number of vessels, whereby the umbilical cord length depends on how much umbilical cord actually remains on the placenta after delivery for postnatal fetal blood sampling.

The placenta itself was weighed without the membranes and cord, and measured and examined for macroscopic abnormalities. For the microscopic examination, the placenta was lamellated and cut into 0.5 cm thick slices. Four blocks were taken. In the first block, a membrane roll, three transverse sections of the umbilical cord near the fetus, the central part and the part near the maternal surface were taken. The other three blocks each contained a full section of the placental parenchyma, including the fetal surface, the placental parenchyma and the maternal surface. The placenta samples should contain both lesion-free tissue and possible lesions. If necessary, more than just the three placental sections can be taken.

Twin placentas were checked for chorionicity and, if dichorionic, the placentas were separated; each placenta was examined separately as if it was a singleton placenta. In the case of monochorial twin placentas, the two territories of the placenta were documented and examined. After the histopathological examination, a detailed histopathological report was prepared containing macroscopic and microscopic findings, as well as a clinicopathological correlation.

## 3. Results

A total of 236 placentas were examined in this study. These consisted of 73 (30.9%) placentas from singleton pregnancies and 54 (22.9%) placentas from twin pregnancies with clinical indications. The control group of 109 (46.2%) placentas included 39 (16.5%) placentas from singleton pregnancies and 70 (29.7%) placentas from twin pregnancies with no clinical indications.

The study included women between 15 and 50 years of age. The mean age at delivery was 32.05 years in the group of singleton placentas with indications and 32.62 years in the control group. The mean age at delivery in the group of twin pregnancies with clinical indications was 33.65 years, while in the control group it was 34.76 years. The gestational age of the pregnancies studied was between the 14th and 43rd gestational week. Of the 236 placentas, 103 were sent with a fully completed request form, while 133 submission forms were incomplete.

### 3.1. Analysis of the Clinical Indications Provided for the Placenta Cohort with Clinical Indications

**Figure 1:**
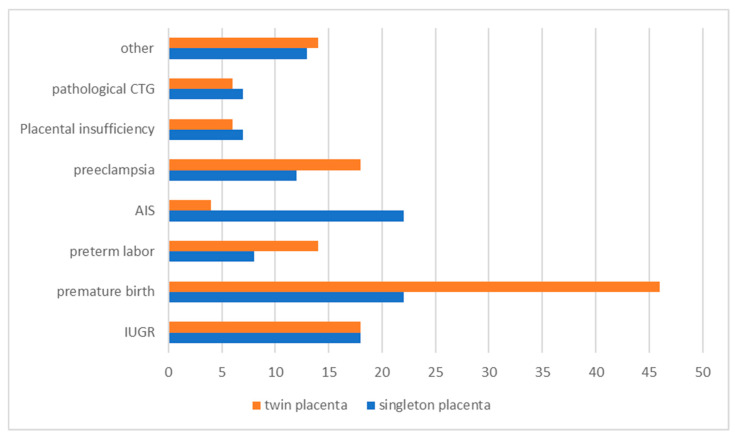
Clinical diagnoses and indications taken from the request form for singleton and twin placentas. (AIS = amniotic infection syndrome, IUGR = Intrauterine growth retardation).

In the group of singleton placentas, a total of 110 clinical indications were given (Figure 1), which corresponds to 150.7%, as there was at least 1 clinical indication for each placenta. The most common reasons for referral were amniotic infection (20%) and prematurity (20%). The second most common reasons were intrauterine growth retardation (16.4%) and pre-eclampsia (10.9%). Other reasons were cited less frequently, including pathologic CTG and placental insufficiency (6.4% each).

A total of 126 clinical indications were given for the twin placentas (Figure 1), which corresponds to 121.2%. Here, too, at least one reason for delivery was listed for each placenta. The most common reasons were prematurity (36.5%), followed by pre-eclampsia and intrauterine growth retardation (14.3% each). Premature labor and other reasons were cited by 11.1% each, while pathological CTG and placental insufficiency were more common (4.8% each) than amniotic infection (3.2%).

A comparison between the two groups of singleton and twin placentas shows that certain reasons for delivery, such as pathological CTG, placental insufficiency, intrauterine growth retardation and others, were similarly frequent in both groups. However, there was a significant difference in the number of reasons for delivery per placenta, with twin placentas having on average more reasons for delivery than singleton placentas. Prematurity was significantly more common as a reason for delivery in twin placentas than in singleton placentas.

### 3.2. Analysis of the Pathology Findings in Both Placenta Cohorts

Similar findings were reported in the histopathology reports of singleton and twin pregnancies. In the group of singleton placentas with clinical indications, a total of 131 diagnoses were reported, which corresponds to 1.79 diagnoses per placenta. In the control group, there were 49 diagnoses, resulting in 1.26 diagnoses per placenta.

Among the diagnoses of the singleton placentas with clinical indications, maternal placental malperfusion (30 = 41.1%) was the most common diagnosis, followed by placental villous maturation disorder (25 = 34.2%) and amniotic fluid infection (20 = 27.4%). Distal villous hypoplasia was mentioned in 16 (21.9%) placentas, while low placental weight occurred in 11 (15.1%) placentas. Villitis/intervillositis was found in only five (6.8%) placentas. Ten (13.7%) placentas had no detectable histopathology, while sixty-three (86.3%) placentas had at least one pathology.

In the control group, 29 (25.6%) placentas showed pathological changes, which were distributed among various diagnoses. In six (15.4%) placentas, no correlation/reporting could be made due to missing information in the pregnancy history.

In the group of twin pregnancies with clinical indications, 54 twin placentas (=22.9%) were examined, which corresponds to 108 individual placentas (monochorial twin placentas were considered as two individual placentas each). A total of 196 diagnoses were mentioned in the report, resulting in 1.81 diagnoses per twin placenta.

The most common diagnosis was placental villous maturation disorder (54 = 50%), followed by maternal placental malperfusion and histologically unremarkable placentas, which were mentioned 40 (37%) and 39 (36.1%) times, respectively. The categories of placental weight discrepancy (20 = 18.5%) and other pathologies (16 = 14.8%) followed. Other diagnoses, such as anastomosis/unequal vascular territories, distal villous hypoplasia, amniotic infection, low placental weight and villositis/intervillositis, were mentioned less frequently.

Of the twin placentas with clinical indications, 39 (36.1%) had no pathologic findings in the report, while 69 (63.9%) had pathologic diagnoses.

In the control group, 70 twin placentas (=29.7%) were examined, which corresponds to 140 individual placentas. A total of 180 diagnoses were mentioned in the report, resulting in 1.29 diagnoses per twin placenta. A total of 96 (68.6%) placentas were histologically normal, while 44 (31.4%) had pathological changes. Placental villus maturation disorder was mentioned in 32 (22.9%) placentas in the report, and maternal placental perfusion was mentioned 16 times (11.4%). Other previously mentioned pathologies were mentioned less frequently.

A comparison of the groups of singleton placentas with the groups of twin placentas shows similar numbers of multiple mentions of pathological findings in the groups with a clinical indication (singleton placentas at 1.79 vs. twin placentas at 1.81) and in the control groups (singleton placentas at 1.26 vs. twin placentas at 1.29). In both the singleton placenta and twin placenta groups, more pathologies were found in the clinically abnormal groups (singleton placenta = 86.3%, twin placenta = 63.9%) than in the respective control groups (singleton placenta = 25.6%, twin placenta = 31.4%), which is statistically significant (*p*-value < 0.05).

There is a clear difference between the diagnoses of singletons and twins in the categories. Twin placentas are more likely to have a placental villous maturation disorder, not only in the group of clinically conspicuous placentas, but also in the control group compared to singleton placentas. In addition, the probability of finding a pathological change in a singleton placenta with a clinical indication is higher than in a twin placenta with a clinical indication. The twin pregnancies are usually scheduled delivered between 34 and 38 weeks of gestation.

### 3.3. Correlation between the Clinical Indications for Histopathologic Examination of Placentas and the Actual Histopathologic Findings

**Figure 2:**
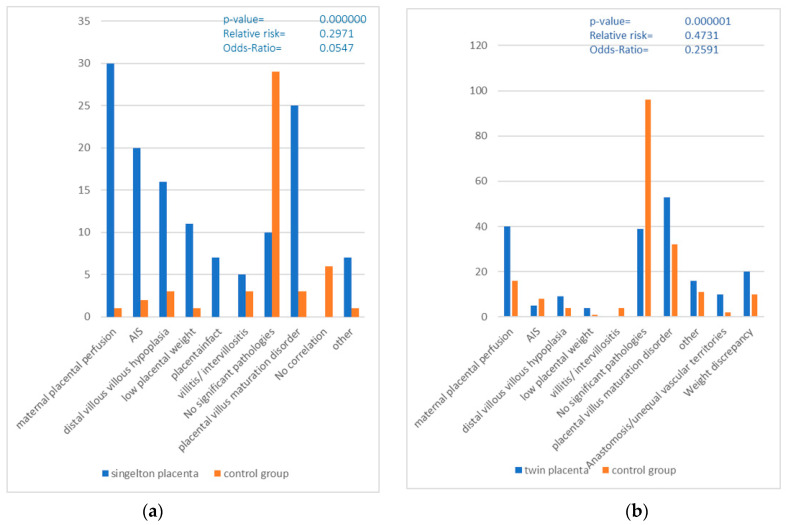
(**a**,**b**) Correlation between the of pathological Placentas and the clinically healthy control group (AIS = Amniotic Infection Syndrome).

**Figure 3:**
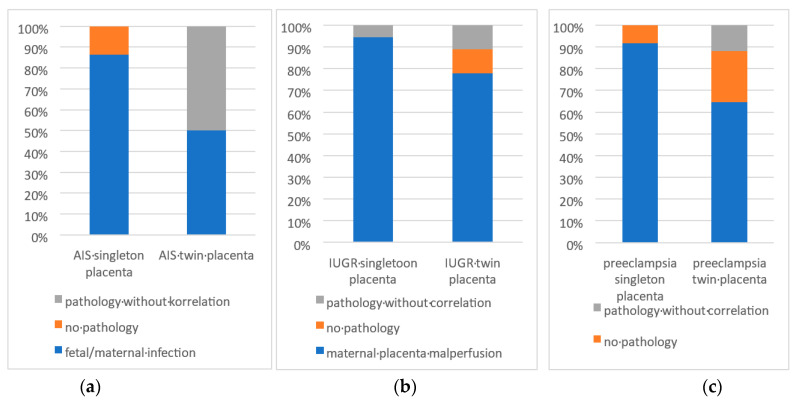
(**a**–**c**): Various histologic findings divided into (**a**) matching the clinical suspected diagnosis, (**b**) other histologic findings deviating from clinical diagnosis and (**c**) no pathologic findings.

1. Singleton placentas with clinical indications:

A high concordance between the clinical indications and histopathologic findings was observed, especially for intrauterine growth retardation (17 out of 18, 94.4%, see Figure 2 and Figure 3a–c), amniotic infection (19 out of 22, 86.6%) and pre-eclampsia (11 out of 12, 91.7%). This suggests that the clinical indications provide reliable clues to the underlying histopathologic changes.

2. Twin placentas with clinical indications:

Compared to singleton placentas, twin placentas showed less concordance between the clinical indications for referral and histopathologic findings, especially for intrauterine growth retardation (14 out 18, 77.8%, see Figure 3a–c), amniotic infection (two out of four, 50%) and pre-eclampsia (11 out of 18, 61.1%). This suggests that the interpretation of histopathologic findings in twin placentas may be more complex or that other factors play a role.

3. Control groups without clinical indications (see Figure 2):

The singleton placentas examined without a specific clinical indication, a total of 25.6%, showed pathological findings. Of these, 15.4% showed pathologies that could be associated with the categories examined (intrauterine growth retardation, amniotic infection or suspected amniotic infection and pre-eclampsia). This indicates that histopathological changes can also occur in placentas without recognizable clinical indications of certain complications. Among twin placentas without specific clinical indications, 31.4% had a pathologic change. Of these, 20% showed pathologies that correlated with the categories examined. This suggests that, even in twin placentas without obvious clinical signs, histopathologic findings can be detected and are potentially less specific if no clinical information is given.

## 4. Discussion

Despite the continuous improvement in prenatal care and antenatal care worldwide, the morbidity and mortality of newborns (4 per 1000 live births [17]) and mothers (4 per 100,000 births [17]) shortly before, during or shortly after birth [17,18,19,20,21,22] in Germany has remained constant since the early 2000s.

These are often preventable causes that can be avoided through better care during pregnancy, during birth and in the postnatal period. Systematic workup, especially through the placenta, could clarify unfavorable pregnancy outcomes and then consider preventive measures for subsequent pregnancies. Placental examination can often provide reliable indications of what led to fetal death, perinatal fetal stress or intrauterine growth retardation (IUGR). A placental examination can also be clinically and medicolegally helpful for poor maternal prepartum and postpartum outcomes, including peripartum deaths.

Although there has been a steady decline in neonatal mortality since 1990 and a global reduction of 50% in neonatal mortality was recorded between 1990 and 2019, this trend has no longer been demonstrable since 2000. Above all, the decline in mortality in the neonatal period is increasingly slowing, which is also due to the changing demographics of pregnant women in terms of the age of first-time mothers and obesity. Even in Germany, some maternal and infant deaths cannot be avoided despite very good prenatal care, although the causes are often preventable or treatable. By submitting placentas with clinical indications for a histopathological examination, possible treatments for the mother and newborn can be initiated more quickly and specifically, recurrence risks for subsequent pregnancies can be identified and better care can be provided or even avoided if necessary.

We were able to show that placentas with pathological pregnancies or births regularly have pathological placental findings that require treatment and/or should be observed preventively in the next pregnancies. We were also able to show that, in the control groups with normal pregnancies or births, there were almost no pathological findings in the placenta. This study shows how important the introduction of a standardized submission form of placentas for histopathological examination is in Germany. This would complement the very good prenatal and postnatal clinical obstetric care in Germany.

It was shown that a standardized request form for placental examination, which is based on established international guidelines [1,2,3,4,5,6], is a good tool in clinical pre-selection to identify indications for placental examination.

A direct comparison of the pathological findings of both groups of placentas with a clinical indication and placentas without a clinical indication once again made this fact clear. As already shown above, pathological changes can be found in the majority of placentas with a clinical indication for a histopathological examination, both in the group of singleton placentas and in the group of twin placentas. In contrast, hardly any pathological changes were detected in the histopathological workup in the respective control groups.

It can be seen that the pre-selection of placentas using a standardized request form in the group of placentas from singleton pregnancies showed a higher correlation between the clinical indication for a histopathological examination and the actual pathological findings. Thus, significantly more pathologies were detected in the group of singleton placentas with a clinical indication than in the group of placentas from twin pregnancies with a clinical indication. There may be several reasons for this. On the one hand, it may be due to the fact that, in the control group, twin placentas with the indication of a premature birth were sent in, which, according to the gestational age, corresponded to a premature birth, i.e., they were born before the 37th week of pregnancy. Strictly speaking, these placentas usually have no histomorphological findings, as both mother and child were healthy, but, in this study, these cases were nevertheless included in the statistics, as they were placentas from probably healthy pregnant women with an unremarkable birth and unremarkable postnatal course in child and mother. For example, placental villi from twin placentas with prematurity often show a villous maturity disorder or a villous maturity that corresponds to a placenta of the late third trimester. Maternal placental malperfusion [8,14], which was detected in some of the control group of twin placentas, can also be associated with prematurity or multiple pregnancies. In these cases, however, they often have no pathological value for the newborn, but are only seen as histopathological findings under the microscope.

This can also be recognized when looking closely at the pathology reports. For each twin placenta, at least two reasons were given on the submission form for the histopathological examination. In addition, prematurity was listed as a reason for submission in almost half of the twin placentas. This leads to the conclusion that prematurity in twin placentas alone is probably often not sufficient to justify a histopathological examination of a twin placenta. Other studies [9,15,23] have already shown that prematurity is more common in twin pregnancies than in singleton pregnancies.

The introduction of a standardized request form is supported by the improved correlation and classification of pathological findings with the clinical information on the submission form. The clinician can thus obtain a correlation of the histological findings with the clinical information from the pathologist. In pre-eclampsia, for example, maternal placental perfusion, distal villous hypoplasia or placental villous maturation disorders are more frequently seen [5,8,14,15]. If intrauterine growth retardation is indicated on the submission form, the histopathological examination often reveals maternal placental malperfusion, distal villous hypoplasia, placental villous maturation disorder and, more rarely, chronic villitis and histiocytic intervillositis, which can have a risk of recurrence [5] of up to 10–17%. By indicating the probability of a possible risk of recurrence in further pregnancies, a faster and more individualized treatment concept can be initiated. In addition, the presence of vascular anastomoses or the presentation of an uneven distribution of vascular territory in monochorial twin placentas can explain the presence of potential fetal growth differences.

In this study, the comparison of the correlation between the reason for referral and the respective findings in the histopathology report showed that the obstetricians were already able to classify the clinical indications very well and were able to identify placentas with a relatively high degree of certainty that had histologically relevant findings. In the majority of placentas, the diagnosis could be confirmed, which can also be important in terms of medical law [9,24], and legitimizes existing therapies retrospectively and prospectively. On the other hand, both in the placentas with indications and in the respective control groups, there was a not inconsiderable number of placentas in which the reason for sending the report did not match the findings of the histopathological report (10 = 13.7% of the singleton placentas with indications showed no pathology, 90.9% of the singleton placentas showed a match between clinical indications and pathological findings; 39 = 36.1% of the twin placentas with indications showed no pathology, with a 62.97% concordance of clinical indications and pathological findings in the twin placentas; 29 = 25.6% placentas of the control group of singleton placentas showed pathology; 44 = 31.4% of the control group of twin placentas showed pathology). The findings that do not correspond to the reason for sending in the placentas further emphasizes the importance of sending in and processing placentas. One of the strengths of this study is the size of the sample, which is comparable with similar studies [25] from other countries on this topic. Another special feature of this study is the presence of a control group consisting of placentas from singleton and twin pregnancies of healthy pregnant women with an unremarkable birth and unremarkable postnatal course in the child and mother. There is no other study on this topic in the literature. The respective control group in the singleton and twin placentas not only allows for assumptions to be made regarding the frequency of the presence of pathologies, but it can also be shown in a direct comparison that the pathological pregnancies and births show pathological findings with significant regularity, which can be important for more individualized and faster treatment for the child and mother. The introduction and use of a standardized placenta request form for histopathological examination provides clinicians with guidance on when a placenta should be submitted.

In addition, the request form provides the pathologist with important information required for a more accurate correlation between the placental pathology and the clinic. On the basis of a well completed request form, the pathologist can prepare a report that is easier for the clinical colleague to interpret. The histopathological examination can provide information on the risk of recurrence in subsequent pregnancies and help to explain pregnancy complications. For the parents, with clinical guidance, the histopathology findings can make it easier to understand why, for example, treatment had to be initiated for the newborn or why the pregnancy/birth had this or that outcome.

## 5. Conclusions

In the majority of placentas that showed a clinical indication for a histopathological examination according to the request form, pathology could be detected. The introduction of a standardized request form, which is based on established international guidelines, can increase the clinical indication for the submission of placentas and the relevance in the clarification and diagnosis of pathological pregnancy outcomes. By listing possible reasons for submitting a placenta, the request form is a good guide as to when a placenta should be sent to the pathology department. In addition, a carefully completed submission form (stating the date of delivery, gestational age, birth weight, maternal BMI, pregnancy history and APGAR score) is an important basis for the histopathological examination and the preparation of a histopathological report, which is easier for the clinical colleague to interpret. On the basis of the submission form, the pathologist can better classify pathological changes, provide explanations for pregnancy complications or assess the risk of recurrence. In addition, individualized treatment management for mother and/or child and for subsequent pregnancies can be initiated on the basis of a conspicuous pathology finding, which contributes to a reduction in preventable and treatable morbidities and causes of death in newborns and mothers.

## 6. Summary

This study demonstrated that the close collaboration between obstetricians and pathologists, using a standardized request form, could provide very effective identification of placentas with clinical indications for histopathological examination. This was validated using a large control group with healthy pregnancies and infants. The introduction of a standardized request form for histopathological examination thus serves as a helpful tool for obstetricians when submitting placentas and, if carefully completed, forms a good basis for the clinicopathological correlation of a histopathological report that can be utilized in the clinical management of the child and mother, as well as in medical–legal contexts and, especially, in quality management. With good communication between clinicians and pathologists, aided by appropriate clinical information provided via a placenta request form, the information obtained from the histological examination of the placenta serves as a valuable supplement and aid in clinical practice.

## Data Availability

The data presented in this study are available on request from the corresponding author.

## Appendix A. Clinical Request Form for Submitting the Placenta

**REQUEST FORM FOR PLACENTA HISTOPATHOLOGICAL EXAMINATION**					
**PATIENT’S DETAILS:** (Alternatively attach patient’s label)		**CLINICAL INFORMATION:**			
**Surname:**		**Date of delivery:**			
**Name:**		**Gestation at birth** (weeks):			
**DOB:**		**Sex:**			
**NHS:**		**Birth Weight (g):**			
**Trust No:**		**Maternal BMI:**			
**Hospital:**		**Obstetric history (G P):**			
**Consultant:**		**Apgar score** (1st min/5th min/10th min)		/ /	
Please note that correct clinical information is essential for an adequate interpretation of placental examination					
**INDICATION FOR PLACENTAL EXAMINATION (please tick ALL relevant):**					
	**Pregnancy loss:**				
	Spontaneous miscarriage (pregnancy loss <24 weeks gestation)				
	Missed miscarriage (incidental finding of pregnancy loss <24 weeks gestation)				
	Stillbirth (pregnancy loss ≥24 weeks gestation)				
	TOP—Indication for TOP:				
	**Intrapartum death/Early neonatal death**				
	**Intrapartum death** (death occurring during labour and baby born with no signs of life)				
	**Early neonatal death**—Indicate age at death:				
	**Severe fetal distress**				
	**Unexpected admission to Neonatal Unit**				
	**Prematurity**				
	**IUGR** (Intrauterine Growth Restriction)				
	**Hydrops fetalis**				
	**Clinical chorioamnionitis**				
	**Abruption**				
	**Morbidly adherent placenta**				
	**Pre-eclampsia**				
	**Multiple pregnancy**:				
	Indicate CHORIONICITY: Indicate CORD IDENTIFICATION [Twin 1: Number of umbilical clips:………………] [Twin 2: Number of umbilical clips:………………]				
	**Any other indication** (specify):				
**TO BE COMPLETED BY DELIVERY SUITE STAFF**. Request form and specimen checked by:					
Print name:			Signature:		Date:
